# Open access – is this the future of medical publishing?

**DOI:** 10.3325/cmj.2013.54.315

**Published:** 2013-08

**Authors:** Hrvoje Barić, Dora Polšek, Lidija Andrijašević, Srećko Gajović

**Affiliations:** Croatian Medical Journal

*“All men by nature desire knowledge“* Aristotle, Introduction to Metaphysics

The *Croatian Medical Journal (CMJ)*, as a small journal that is not part of any publishing group faces serious challenges to prosper in a multitude of diverse biomedical journals. In such a complex situation, adaptations are always necessary. They include changing the editorial policy, embracing new ideas, and introducing new members to the Managing Team. Besides selecting the right articles, expert reviewers, and collaborators, the question of the mode of access to the published articles was recognized as one of the most important for the future of the *CMJ*.

The *CMJ* is an open-access journal with full texts freely available on both the journal’s Web site (*www.cmj.hr*) and on PubMed Central (*http://www.ncbi.nlm.nih.gov/pmc*/). Managed by four Medical Schools in Croatia, it is funded through institutional resources and by annual grants from the Croatian Ministry of Science. Furthermore, the *CMJ* has a contract with the publisher “Medicinska Naklada,” who covers publishing and printing costs, and shares the profit or loss with the Journal (through the years a modest profit has always been present, with no losses). This agreement was officially and legally defined in 2003 (1). When a few years ago two major publishing companies approached the *CMJ*, the decision was not to join them, despite being aware that the *CMJ* is a rather small journal from a small country (2). From the very beginning, the *CMJ* has insisted on being an open-access journal, not charging fees to either authors or readers and providing no compensation to reviewers. This model is sometimes named diamond or platinum open access (OA), and we are very proud to maintain it as a major feature of the *CMJ*. Still, in view of the recent economic crisis, with institutional and governmental funding being rigorously reduced, an important question arises – is this business plan idealistic or sustainable?

## Open access as a necessity

The initiative for OA appeared in the second half of the 20th century and gained momentum with the appearance of the internet. In the late 1980s and early 1990s, the scientific community witnessed the emergence of the first free open access peer-reviewed journals (OAJs) and free scientific online archives (3). In 1994, when the internet was becoming widely available, Stevan Harnard started an initiative to make research broadly accessible to the public (4). Almost twenty years later, the significance and impact of OA is still under debate, with many questions about its organization and implementation being raised.

OA means unrestricted access to peer-reviewed scholarly articles, books, and other publications, and such publishing has in recent years achieved stronger growth than the conventional subscription-based publishing, as well as reached the same level of quality and impact in some research fields such as medicine and health (5,6). However, in an intricate web of profit and priorities the implementation of this fundamental concept is a significant challenge, attempting to reconcile publishing fees with research and subscription funding to keep scientific publishing a profitable economic activity. The basic idea of providing unrestricted online access to scholarly articles has developed into different modes of providing OA. Depending on the uttermost provider of articles, OA is divided into green and gold OA and is furthermore categorized into subtypes by sources of funding (7).

**Green OA** enables free access to articles by authors communicating their findings through institutional or central repositories like PubMed for example, or by simply depositing peer-reviewed postprints (revised by authors or the publisher`s version) on other OA web pages, a practice known as “self-archiving.” The Registry of Open Access Repositories (ROAR) includes over 2000 institutional repositories, while specialized search engine services like Google Scholar and OAIster are dedicated to finding specific manuscripts (8) (*http://scholar.google.hr*; *http://oaister.worldcat.org/*). Although self-archiving initiative often comes from the authors themselves as a statement of good-will, more and more research funders and institutions mandate that research they support should be publicly available in order to ensure the impact of the invested funds (8). The Registry of Open Access Repository Mandatory Archiving Policies reported a considerable increase in the number of institutions that mandate archiving for their researchers, amounting to 173 universities applying this policy in 2013, including the research funded by the European Commission, Harvard, and Massachusetts Institute of Technology (8).

**Gold OA** is a mode of publishing through OAJs, where articles are made available on the publisher’s Web sites, prominent examples of such biomedical publishers being BioMed Central and PLoS. OAJs may charge a publishing fee for authors or their funders or may waive the fees completely.

Since the beginning of the OA initiative, when only two “trends” of OA were defined, a whole range of variations in the development of the OA concept has emerged, leading to a considerable confusion and misuse of different terms. In the need for a more precise terminology indicating different business models for OAJs, the term “platinum” or “diamond” OA has come to indicate an OAJ that does not charge any fees to contributors or readers alike (9). While some experts think new terms further complicate the OA debate (10-14), we consider that the distinction between journals that charge fees and those that do not is important enough to merit a clear distinction. There are currently 9759 journals registered in the Directory of Open Access Journals, while 6567 of those do not charge processing fees (*www.doaj.org*).

## A paradigm shift

From 2000-2009, there has been a 10-fold increase in the number of journals that follow the gold OA policy (15). A study from 2013 estimated that 7.9% of all peer reviewed scientific articles were published in OA journals (16). The percentage of overall data made available by OA and the mode of publication of the results vary in different fields of research. Medical publishers “prefer” gold OA, mathematical and physical green OA with individual self-archiving (17). In 2009, 21.7% of medical publications were openly accessible, 13.9% of through OAJs (17). A recent study from 2013 reported a “remarkable growth of open access in the biomedical field” – claiming that more than 50% of the biomedical articles were published in OAJs (18). The increase in OA implementation is also seen in a higher portion of peer-reviewed articles found in OA repositories, share of OA journals with high impact factors, etc (17).

While the data showing upward trends are reassuring, there is an on-going debate whether OA journals are really beneficial for the scientific community. The main argument given by OA advocates is that through the process of promoting transparency and sharing, the researchers as well as the general public benefit from widely available knowledge that would otherwise be restricted to “privileged” individuals and institutions. Furthermore, they present evidence that OA leads to more citations, article quality improvement, and acceleration of research progress, productivity, and knowledge translation (19-21). The availability of information promotes the translation of published research into good practice. Particularly in medicine, family practitioners and specialists, who frequently do not have access to recent findings through institution-paid subscriptions, are able to integrate new knowledge into their everyday work without investing resources to access the needed content. Other end-users, such as patients and laymen, benefit as well, since open access publications can help deconstruct the notion that research papers are intended for researchers only (19). The major potential of OA, especially in medical practice, is in the improvement of health care quality through rethinking and re-defining the established approaches.

Although the body of evidence supporting OA is strong, critics argue that by itself, the concept does not provide solutions, it simply alters the path of financing (22). The funding of OA journals is a complex puzzle in which all participants are expected to work together in order to achieve the long-term vision of making all published articles openly accessible. Currently, one third of OA journals charge publishing fees, while others receive institutional, governmental, or third-party funding (23). In theory, if institutions were not compelled to pay high subscription fees for non-OA journals, they would have “free” funds for paying publishing fees and dissemination of their work (24). One of the rare downsides of OA is the emergence of predatory publishing, where journals in order to profit from publishing fees accept articles with no peer review and little quality control. This practice has harmed the OA mission, and brought the sustainability of the OA model into question (25). As a reaction to predatory publishing, the Open Access Scholarly Publishers Association was formed to promote and authenticate a positive public image of OA publishing (6).

In case of transition from subscription-based system to OA publications, the focus must be kept on increasing availability, questioning exclusivity, and preventing recirculation of funds, knowledge, and quality in the same exclusive community of the most respectable journals and institutions. The *CMJ* is aware of the impact that the OA has on health care today, and it is dedicated to provide full accessibility of its publications without processing fees, in the hope that everyone will benefit from our efforts to disseminate knowledge.

Still, as we maintain OA without charging the processing fees to the authors, the question remains – *Who pays for the published articles?* As there is no “free lunch” anywhere, especially not in science publishing, this has been one of the crucial issues to consider for future sustainability of our journal. We still depend on institutional and governmental funding, which helps to maintain and develop our activities. This finally means that neither authors nor readers have to pay, but that the *CMJ*, a global medical journal, is supported by the Croatian tax-payers. We argue that this indeed is in their interest, because this makes Croatia a place where important knowledge of managing a global scientific journal is independently generated and maintained.

In the hope that this public Croatian support is sustainable, we consider the desire to put quality over profit as feasible and realistic. Through this effort, although small, the Croatian scientific community will continue to benefit from an unbiased, objective, open-access journal with high criteria for both authors and reviewers.
